# Morality in middle childhood: the role of callous-unemotional traits and emotion regulation skills

**DOI:** 10.1186/s40359-023-01328-7

**Published:** 2023-09-21

**Authors:** Jessica Wilke, Naska Goagoses

**Affiliations:** Department of Special Needs Education & Rehabilitation, Carl von Ossietzky Universität of Oldenburg, Ammerländer Heerstraße 114-118, 26129 Oldenburg, Germany

**Keywords:** Morality, Callous-unemotional traits, Emotion regulation skills, Middle Childhood

## Abstract

**Background:**

The development of morality is vital for fostering prosocial behavior and enhancing both individual and societal well-being. Clarifying what contextual and individual factors play a role in moral processes during childhood can contribute to our understanding of the development of morality. Given the previous acknowledgment of importance, yet lack of existing empirical findings, the study focused on the significance of callous-unemotional traits (i.e., an affective-interpersonal personality trait, related to psychopathy in adulthood) and emotion regulation (i.e., an essential part of socio-emotional competence, and a transdiagnostic factor in the development of psychopathology) for moral emotions and cognitions during middle childhood. The concrete aim was to examine direct and indirect effects of callous-unemotional trait dimensions (callousness, uncaring, unemotionality) onto immoral emotional attribution (i.e., feeling good after immoral decisions) and admissibility of immoral actions (i.e., evaluating immoral actions as being okay) via emotion regulation skills.

**Methods:**

A cross-sectional study was conducted with 194 children attending Grades 1 to 4, and their primary caregivers. The children completed the Inventory of Callous-Unemotional Traits and caregivers completed the Emotion Regulation Checklist. The children were also presented with a set of moral dilemma vignettes, and asked about the emotions of protagonists who acted immoral, and the admissibility of their actions.

**Results:**

Path-model analysis revealed (1) negative direct effects of emotion regulation skills onto immoral emotional attribution and admissibility of immoral actions, (2) positive direct effects of the dimensions callousness and uncaring onto immoral emotional attribution and admissibility of immoral actions, and (3) negative direct effects of dimensions callousness and uncaring onto emotion regulation skills. Indirect effects, indicating that emotion regulation skills mediate the association between the callous-unemotional trait dimensions and morality, were also found.

**Conclusion:**

The findings address a knowledge gap and indicate that emotion regulation skills, callousness, and uncaring play an important role in morality in middle childhood and should be included in frameworks of moral decision-making and development.

## Introduction

Morality comprises the concept of distinguishing right from wrong, guiding individuals’ actions and interactions within society [1]. In middle childhood, essential prerequisites for moral actions (e.g., perspective-taking skills, theory of mind, emotional understanding, behavioral regulation) are present [2, 3]. These foundational aspects are intertwined with behavior, where morality profoundly shapes both society and individuals. Morality underpins norms and rules that govern interactions. However, it’s vital to recognize the lack of universal consensus on moral definitions [1]. In research, prosocial and positive developmental outcomes are associated with morality. Children with strongly internalized moral values are more prosocial and caring, whereas children with poor internalization of moral values often exhibit antisocial behaviors and aggression [4]. Given the significance of morality for both the individual and the community at large, research has focused on identifying both contextual factors (e.g., relationships, culture) [5, 6] and individual factors (e.g., personality, emotions) [7, 8] that influence moral processes and the development of morality. Emotion regulation skills and callous-unemotional traits have both been theoretically identified as significant individual factors for morality [7, 9, 10], yet very little empirical work examining the significance of emotion regulation and callous-unemotional traits for morality in childhood exists [11, 12]. Considering that callous-unemotional traits have also been linked with emotional competencies [13, 14], a simultaneous examination can offer insights into the mechanisms involved in moral decisions and actions through cognitive and emotional morality.

The aim of the current study is to examine the association between callous-unemotional traits, emotion regulation skills, and morality in middle childhood. We postulate a theoretically- and empirically-driven integrative mediational path model between the constructs. We first examine hypothesized direct associations between (1) callous-unemotional trait dimensions (callousness, uncaring, unemotionality) and emotion regulation skills, (2) emotion regulation skills and cognitive and emotional morality, and (3) callous-unemotional trait dimensions and cognitive and emotional morality. We then examine hypothesized indirect effects in a mediational path model.

## Theoretical background and literature review

### Morality: moral emotions and cognitions

Moral psychology has a long tradition and distinguishes various constructs of morality (e.g., moral emotions or judgment). Discussions about the concept of morality and the moral development of children span across diverse theoretical perspectives and models. These perspectives and models encompass distinct foundations (such as emotional and cognitive perspectives) and employ varied operationalizations of morality [12, 15]. In response to this diversity, Oser [1] developed a comprehensive and integrative model of moral motivation. He attempts to consolidate diverse perspectives into a unified model. Within this model, he contends that the central moral self (internalization of moral values) activates moral judgments and vision, which in turn influence individual beliefs, moral emotional attributions, moral motives and interests; these constructs then lead to moral deliberation, determination of one’s responsibility or judgment of admissibility, and sense of duty, and lastly, moral actions ensue. Oser’s model [1] highlights the need to consider multiple constructs of morality and especially look at both emotional and cognitive moral constructs to understand morality.

Emotional attribution is a component of moral motivation, i.e., an emotional moral construct [1], and encompasses feelings and emotional responses attributed to moral/immoral decisions [16, 17]. In moral transgressions, two distinct types, based on the nature of experienced emotion (happy or unhappy), can be distinguished [18]. Happy victimizers exhibit positive or neutral emotions during immoral decisions, whereas unhappy victimizers experience guilt or negative feelings in response to immoral decisions [18]. Consequently, immoral emotional attribution is the assignment of feelings to immoral decisions, involving positive or negative emotions after transgressions. These attributed emotions in moral transgressions reveal a child’s alignment with moral principles. For instance, children attributing negative emotions to immoral actions demonstrate commitment, whereas those attributing positive emotions to immoral actions exhibit a lack of commitment to moral principles [12, 19].

The perceived admissibility of immoral actions is part of moral cognition, i.e., a cognitive moral construct [20, 21]. Admissibility of immoral actions is defined as the evaluation that immoral actions are acceptable or permissible, i.e., evaluating moral actions as not obligatory, not worth the personal costs, and that immoral actions are allowed as an exception [22, 23]. The evaluation of the perceived admissibility of moral transgressions is influenced by moral knowledge of right and wrong as well as moral norms and rules [20, 23].

Perceptions and information processing play an important role in moral conflict situations [10]. When confronted with moral conflicts, children anticipate potential feelings of moral or immoral action and evaluate whether a moral transgression is admissible or not [10]. As such, immoral emotional attribution and admissibility of immoral actions can be viewed as components of social information processing in moral conflicts. Garrigan et al. [10] proposed that morality depends on various factors that influence different steps in the process and overall moral development; these include cognitive (e.g., perspective taking, schemas), social (e.g., social skills, peer interactions), and affective components (e.g., empathy, emotion recognition, emotion regulation). Importantly, these cognitive, social, and affective components are linked; for instance, empathy can only motivate moral actions, when individuals possess adequate emotion recognition and regulation skills, which in turn are influenced by genetic predispositions of emotionality or temperament/personality [10]. In the current study, we examine emotion regulation skills and callous-unemotional traits as potential components that influence immoral emotional attribution and admissibility of immoral actions in moral situations.

### Emotional regulation skills and associations with morality

Emotion regulation is a core aspect of emotional competence [24], consisting of processes to maintain, monitor, evaluate, modify, or inhibit emotional reactions to accomplish one’s goals [25, 26]. Children acquire different emotion regulation skills and strategies [27], which influence their further social development. Furthermore, scholars have highlighted the importance of emotion regulation skills for moral development [7, 10, 28, 29]. In this context, special attention is paid to the role of the moral emotion empathy [7, 10]. To feel empathy within moral situations, individuals must first have the ability to correctly recognize others’ emotions, regulate their own emotions, and retrieve relevant connections between empathy and cognition from memory [10]. Previous studies have also linked emotion regulation skills to constructs like moral emotions [30], moral reasoning [31], and general moral skills [32] in childhood; with a meta-analysis of these studies showing that emotion regulation skills have a medium effect on emotional and cognitive aspects of morality within childhood [33]. Overall, the results show that increased emotion regulation skills resulted in better moral outcomes (e.g., higher empathy) [30]. If negative emotions are not appropriately regulated in moral situations, they can disrupt moral cognition [31]. For example, high empathic arousal in a moral conflict can lead to personal distress. To regulate this distress, many cognitive resources are used, so that individual needs are focused and moral reflection is inhibited [30, 32]. Distinguished and adaptive emotion regulation skills can therefore contribute to acting morally in the context of moral conflicts [33].

### Callous-unemotional traits and associations with morality

Children with callous-unemotional traits have been described as lacking guilt and empathy, as well as using others for their own gain and expressing shallow emotions [34]. Callous-unemotional traits encompass the dimensions *callousness*, i.e., a lack of remorse and concern about others, *uncaring*, i.e., not caring about others or own performance, and *unemotionality*, i.e., not expressing emotions [35]. Callous-unemotional traits are described as a multifaceted affective-interpersonal (personality) trait [36], being a precursor for antisocial behaviors and psychopathy in adulthood [37, 38]. Etiological examinations have found both biological and environmental precursors relevant for the development of callous-unemotional traits; these include genetic heritability [39], pathological neuro-physiological mechanisms [40], deficits in affiliative reward processing and threat sensitivity [41], temperamental fearlessness [42], parental attachment [43], and parenting practices [44]. Based on these factors children may have a biologically-driven deficit in emotional processing that results in reduced sensitivity to cues, or may cope with harsh environments by adopting callous traits and becoming emotionally detached [11].

Drawing a connection between callous-unemotional traits and morality, Frick et al. [9] proposed that callous-unemotional traits may be viewed as the development of conscience gone amiss. Specifically, they note that conscience describes the moral emotions of empathy and guilt, which promote prosocial behaviors (i.e., voluntary behaviors that benefit other people). Frick et al. [9] state that children with callous-unemotional traits may not follow normal developmental pathways, which include the internalization of moral attributions and judgements [22], and increasing guilt and remorse following moral transgressions [45]. Craig et al. [11] note that the deficiencies in social-emotional processing in children with callous-unemotional traits impair their moral development.

Despite the overall connection on a theoretical level, only a few studies have explicitly examined associations between callous-unemotional traits and morality. The callousness and uncaring dimension have been linked with moral identity in adolescents [12], and the overarching scale of callous-unemotional traits has been linked to moral disengagement in preadolescents [46]. Examining moral reasoning, Blair [47, 48] found that children high on psychopathic traits (i.e., callous-unemotionality, narcissism, and impulsivity) were worse at distinguishing between moral and conventional transgressions, and were less likely to attribute moral emotions to protagonists, than children low on psychopathic traits. Furthermore, adolescents with higher callous-unemotional traits report less guilt and less wrongness appraisals of moral transgressions [49]. Lastly, Thornberg and Jungert [50] found that callousness and unemotionality had a direct negative effect on moral reasoning (judging moral transgressions as wrong while focusing the harm of others) in a sample of elementary school children.

### Associations between callous-unemotional traits and emotion regulation skills

Studies examining longitudinal and concurrent associations have shown that callous-unemotional traits are inherently linked with emotional constructs. That is, callous-unemotional traits have been (negatively) linked with various emotional competence skills relevant for middle childhood. For one, overarching callous-unemotional traits were negatively associated with emotional intelligence in a sample of incarcerated adolescents [51]. Furthermore, overarching callous-unemotional traits are negatively associated with empathy [14], even within a community sample of middle school students [13]. Studies have also found that overarching callous-unemotional traits are positively associated with problems in emotion recognition and emotion awareness in middle childhood [13, 52, 53]. Baroncelli et al. [54] found that the unemotionality dimension was associated with a lack of emotion awareness, and the callousness dimension was associated with a lack of attention to the emotions of others in preadolescence. Furthermore, overarching callous-unemotional traits positively correlate with emotion dysregulation during adolescence [43] and adulthood [55], and children with high callous-unemotional traits display more emotion dysregulation than those with low callous-unemotional traits [56]. Lastly, the unemotionality dimension shows disparities in its association with different types of emotion regulation strategies (e.g., a positive correlation with expressive suppression, and no correlation with cognitive reappraisal) [57].

## Current study

Garrigan et al. [10] proposed that moral development, as well as the perception and information processing in moral situations, all of which essentially influence moral behaviors, are dependent on preestablished cognitive, social, and affective schemas and skills. We propose that callous-unemotional traits and emotion regulation skills are part of these components, which influence moral emotions and moral cognitions that form part of the information processing in moral situations. The literature highlights that children with callous-unemotional traits have difficulties with various emotional competencies [13, 52, 53, 56], due to biologically-driven deficits or as a coping response to environmental hardships [11]. As emotional competencies influence information processing in social interactions [58], and also in moral situations [10], difficulties therein negatively impact moral emotions, cognitions, and behaviors [33]. Based on these theoretical models and partial empirical findings, we propose that children with higher levels of callous-unemotional traits will possess lower emotion regulation skills, which in turn will affect immoral emotional attribution and admissibility of immoral actions in moral situations. As children with callous-unemotional traits also feel less guilt or remorse when acting immorally [9], and evaluate moral transgressions as admissible [49], higher levels of callous-unemotional traits will also directly affect immoral emotional attribution and admissibility of immoral actions. The simultaneous consideration of all three constructs adds value to understanding and expanding on the mechanisms involved in moral decisions and actions.

Hence, the overall aim of the current study is to examine the proposed association between callous-unemotional traits dimensions callousness, uncaring, and unemotionality, emotion regulation skills, and cognitive and emotional aspects of morality in middle childhood in an integrative mediational model. Based on previous theoretical and empirical research, the following hypotheses were formulated for the current study: [H1] the callous-unemotional trait dimensions callousness, uncaring, and unemotionality are negatively associated emotion regulation skills (i.e., direct negative effect); [H2] emotion regulation skills are negatively associated with immoral emotional attribution and admissibility of immoral actions (i.e., direct negative effect); [H3] the callous-unemotional trait dimensions callousness, uncaring, and unemotionality are positively associated with immoral emotional attribution and admissibility of immoral actions (i.e., direct positive effect); [H4] emotion regulation skills mediate the association between the callous-unemotional trait dimensions and immoral emotional attribution and admissibility of immoral actions (i.e., indirect effect).

We focus on middle childhood, as it constitutes an interesting developmental stage for morality. In early childhood, children learn moral rules through authorities, e.g., parents or preschool teachers, whereby their orientation is still very rigid and rule-following [59]. In middle childhood, important developmental processes ensue, whereby children increasingly detach themselves from the rules that authority imparts, increasingly internalize moral values for themselves, and advance their moral understanding [59]. In addition, marked advances in social and cognitive development relevant for morality occur during middle childhood [60, 61]. Despite being under-researched, middle childhood is seen as an ideal phase for examining processes in moral development, more so than early childhood or adolescence [61].

In addition, important methodological considerations flow into the current study. Oser [1] highlighted the need for morality to be viewed from both an emotional and cognitive perspectives, thus prompting us to include both an emotional moral construct (i.e., immoral emotional attribution) and a cognitive moral construct (i.e., admissibility of immoral actions). Depending on their study aims, researchers have examined callous-unemotional traits as one overarching construct or separately in its dimensions [35, 62]; the later has often revealed that the dimensions have differential associations with emotion-related and behavioral outcomes [35, 54, 57, 63], indicating that a separate examination may provide useful for understanding moral processes. Lastly, studies indicate that age, gender, and special educational needs can have main effects on callous-unemotional traits [62–66], emotion regulation skills [67–71], and morality [72–76], yet inconsistencies have been recorded. Nonetheless, we opted to add gender, age, and special education needs in emotional-social development as control variables in the path model.

## Methods

### Design and procedure

The data stems from a larger project with the aim of examining the social-emotional development of morality in middle childhood. The current study is a cross-sectional questionnaire study, and we only report on the instruments and data relevant to the current research question. Approval was obtained from the institutional review board and the regional school authority board. Aiming to recruit participants in middle childhood, i.e., ages ranging from six to eleven years [77, 78], students attending Grades 1 to 4 were selected as the target group, as this most closely encompasses the sought age range. Based on convenience sampling, schools in various German regions were contacted and informed about the study. School management was able to voluntarily decide whether they wanted to participate, and whether the data collection should be carried out at the school or online. At participating schools (13 primary schools and 7 special education schools), informed consent letters were distributed to the primary caregivers of students in Grades 1 to 4; participation was voluntary and only possible with signed consent forms from parents or legal guardians. Children provided oral consent. Data collection took place between January and May 2022.

### Participants

The sample consisted of 194 six- to eleven-year-olds (*M*_age_ = 8.53, *SD*_age_ = 1.40; 58.8% male) and their primary caregivers (*M*_age_ = 40.41, *SD*_age_ = 5.94). Primary caregivers were asked to indicate their familial relation to the child (e.g., mother, father, foster parent), and whether they filled out the questionnaire alone or together with someone else (e.g., mother and father together). Of the primary caregivers 75.8% were biological parents (62.9% of questionnaires were completed solely by mothers; 4.1% solely by fathers, and 8.8% by both parents in unison) and 10.3% were other primary caregivers; 13.9% of primary caregivers did not respond. 146 children visited regular primary schools and 48 special education schools; a total of 52 children had a special educational need in emotional-social development status. In Germany a school-oriented diagnostic procedure is undertaken by specialized educators to assign special educational needs. A special educational need in emotional-social development, represents a specific educational need due to emotional and behavioral difficulties. 2.1% of the children and 12.4% of the caregivers were not born in Germany. Caregivers were asked to report the highest level of education completed by both the mother and father. Concerning mothers and fathers respectively, 2.6% and 3.6% did not complete secondary school; 4.6% and 8.8% completed Grade 9; 29.4% and 27.8% completed Grade 10; 12.9% and 11.3% completed Grade 12/13; 26.3% and 22.2% obtained a university degree; 4.6% and 5.2% indicated other; 19.6% and 21.1% left the question unanswered.

Initially, we set out to recruit 300 children. However, as a result of limited resources at schools (due to the covid-19 pandemic and integration of migrant children), we stopped data collection after five months. To determine if the current study was sufficiently powered, a sensitivity analysis using G*Power [79] was conducted. The post hoc test for this sample size (*N* = 194) yielded a power of 92.76%, when an effect size *f²* = 0.05, α err prob. = 0.05, and a number of seven predictors were included.

### Measures

*Callous-Unemotional Traits*. The German-version of the Inventory of Callous-Unemotional Traits [62, 80] was used to measure callous-unemotional traits. This inventory is a psychometrically validated instrument to assess callous-unemotional traits in childhood and adolescence [34, 35, 62]. The inventory includes 24 items (*α* = .85, Ω = .85), which form the sub-scales callousness (e.g., “I do not care who I hurt to get what I want”; 11 items, *α* = .77, Ω = .78), uncaring (e.g., “ I always try my best (reverse scoring)” ; 8 items, *α* = 0.77, Ω = 0.77), and unemotionality (e.g., “I do not show my emotions to others”; 5 items, *α* = 0.73, Ω = 0.73). The participating children were asked to respond to the items on a scale ranging from (1) not at all true to (4) definitely true. Items that required reverse scoring were recoded. Hence, high values per dimension indicate higher levels of callousness, uncaring, and unemotionality.

*Emotional Regulation Skills*. The Emotion Regulation Checklist is an other-report instrument used to assess emotion regulation skills [81], encompassing affective valence, flexibility, intensity, lability, and situational appropriateness [82, 83]. The instrument has already been psychometrically proven in various countries and languages (e.g., Brazil: [84]; Italian: [85]; Persian: [86]; Preschooler in Turkey: [87]). Utilizing a back-translation method, the emotion regulation sub-scale, which focuses on empathy, appropriate affective displays, and emotional self-awareness (e.g., “Can say when he/she is feeling sad‚ angry‚ or mad‚ fearful or afraid”; 8 items, *α* = 0.81, Ω = 0.76), was translated into German. In addition, an exploratory factor analysis was conducted, which yielded a single-factor structure (Bartlett’s test: *χ*^*2*^(28) = 377.68, *p* < .001; KMO = 0.83; *R*^*2*^ = 0.44) with high factor loadings ranging from 0.51 to 0.79. Caregivers were asked to respond to the items on a scale ranging from (1) rarely or never to (4) almost always. Items that required reverse scoring were recoded. High values indicate higher levels of emotion regulation skills.

*Immoral Emotional Attribution and Admissibility of Immoral Actions*. The Moral Attitudes in Adolescence questionnaire [88] was used to assess immoral emotional attribution and admissibility of immoral actions. The questionnaire has shown adequate reliability [12]. The questionnaire was adapted linguistically and visually for children. Additionally, some of the moral conflicts were transformed into moral transgressions. The questionnaire for children includes 4 moral conflicts and 4 moral transgressions stories, designed according to the moral dilemmas of Weller and Lagattuta [89, 90]. The short stories follow the structure and specification of Christensen and Gomila [91], namely: (1) format of presentation, (2) expression, (3) word-framing effects, (4) subject’s perspective, (5) previous description of the situation, (6) order of presentation, (7) type of question, (8) relationship (e.g., ingroup/outgroup, friend or relative), (8) question type, (9) type of transgression, and (10) certainty of the event. The moral conflicts describe a situation in which a protagonist is torn between two incompatible outcomes; for example, choosing between the pursuit of one’s own needs versus the interests of others [90, 91]. The moral transgressions describe a situation in which a protagonist breaks a moral rule; for example, intentionally causing harm to someone else [92]. The stories were designed to closely resemble situations that might be encountered in everyday lives (e.g., helping a peer, lying to a best friend). Each story was accompanied by a visual depiction, with the protagonists’ gender being matched to the participants’ gender.

The following is an example of a moral conflict story presented to girls: “Lisa enters the schoolyard and sees that a girl is hitting Sandra because she sat on the bench that the girl normally sits on. Lisa thinks about what she can do. She knows that if she intervenes and tells the girl to stop, she will be late for class. But, if she goes into the school building, the girl will continue beating Sandra.”. After reading the story, the participants are asked how they think the protagonist would feel if opting for the immoral choice (e.g., “Lisa decides to go into the school building. How good or bad do you think Lisa feels about that?“). The participants are asked to respond on a scale ranging from (1) very bad to (5) very good. This question is indicative of participants’ immoral emotional attribution, with high values reflecting feeling good after an immoral decision (8 items, *α* = 0.86, Ω = 0.86). Not feeling bad after an immoral decision may reflect a lack of guilt or shame. Furthermore, the participants are asked how admissible the immoral action was (e.g., “In picture B, Lisa has decided to go into the school building. How okay do you think it was for Lisa to do this?”). The participants are asked to respond on a scale ranging (1) not okay at all to (5) very okay. This question is indicative of participants’ admissibility of immoral actions, with high values reflecting high permissibility of immoral actions (8 items, *α* = 0.83, Ω = 0.83). As there are eight stories, the immoral emotional attribution and admissibility of immoral actions variables constitute eight items each.

### Data analysis

The data was analyzed with SPSS 27 and AMOS 27. Descriptive and preliminary analysis were conducted first. The callous-unemotional trait dimensions, emotion regulation skills, and immoral emotional attribution and admissibility of immoral actions, are the key variables in the path model. Gender, age and SEN are the control variables. The path model included the dimensions of callous-unemotional traits as independent variables, emotion regulation skills as the mediator variable, and immoral emotional attribution and admissibility of immoral actions as the dependent variables. To control for gender, age and special educational needs, these were added as additional independent variables with direct paths onto all key variables in the path model. Direct effects between the variables were estimated. Indirect mediation effects were determined using a bootstrap method with confidence estimates; a confidence interval at the 95% level with a bootstrap of 1000 samples was used for the estimation [93]. Bootstrapping was utilized as it can compensate for potential deviations from normal distributions [94]. Missing data analysis indicated that they were missing completely at random (MCAR; *χ²* = 83.619, df = 77, *p* = .284) [95]. As the data was MCAR, full information maximum likelihood estimation of the regression coefficient was possible. To assess the model fit the Root Mean Squared Error of Approximation (*RMSEA*), Comparative Fit Index (*CFI*), Normed Fit Index (*NFI*), Tucker-Lewis Index (*TLI*), *χ²*- and *p*-value were examined. An adequate model fit is indicated with the following values: *χ²*/df < 5, *χ²* not significant, *CFI*, *NFI* and *TLI* near 1 (> 0.90), and *RMSEA* and *SRMR* near 0 (< 0.08) [96, 97].

## Results

### Preliminary analyses

Table 1 presents the mean and standard deviation of each variable, as well as the correlations between them. The values indicated that the levels of callousness and uncaring were low to middle in average, as were those for immoral emotional attribution and admissibility of immoral actions. The average level of emotion regulation skills was middle to high, and average values for unemotionality were high. On average, children’s values in immoral emotional attribution and admissibility of immoral actions were low, comprising a bad feeling to immoral decisions and evaluating the immoral action as not okay.

**Table 1:** Intercorrelations, means and standard deviations

	1	2	3	4	5	6
Key Variables						
1 Callousness	1					
2 Uncaring	0.40***	1				
3 Unemotionality	0.43***	0.37***	1			
4 Emotion regulation skills	− 0.49***	− 0.49***	− 0.40***	1		
5 Immoral emotional attribution	0.58***	0.44***	0.29***	− 0.47***	1	
6 Admissibility of immoral actions	0.56***	0.43***	0.37***	− 0.43***	0.88***	1
Control Variables						
7 Age	0.24**	0.16*	0.23**	− 0.16*	0.10	0.10
8 Gender	0.14	0.00	0.08	− 0.15*	− 0.05	− 0.04
9 SEN	0.27***	0.07	0.24**	− 0.33***	0.11	0.07
*M*	1.66	1.79	2.07	3.18	1.97	2.09
*SD*	0.45	0.50	0.60	0.48	0.70	0.67

*Note*. *p < .05; **p < .01; ***p < .001; *M* = Mean; *SD* = Standard deviation; SEN = Special educational need in emotional-social development; Gender was coded with girls = 1 and boys = 2; SEN was coded with 1 = without SEN and 2 = with SEN.

The three callous-unemotional trait dimensions were positively correlated with each other, and with immoral emotional attribution and admissibility of immoral actions, as well as being negatively correlated with emotion regulation skills. Emotion regulation skills were negatively correlated with immoral emotional attribution and admissibility of immoral actions. Age correlated with the three dimensions of callous-unemotional traits and emotion regulation skills, but not with immoral emotion attribution and admissibility of immoral actions. Gender correlated only with the dimension of callousness and emotion regulation skills. Special educational needs in emotional-social development significantly correlated with the dimensions of callousness and unemotionality and with emotion regulation skills. Cronbach’s alpha and McDonald’s omega were calculated,  and indicated sufficient or good reliability for all [98, 99].

#### Path-model analysis

**Fig. 1:**
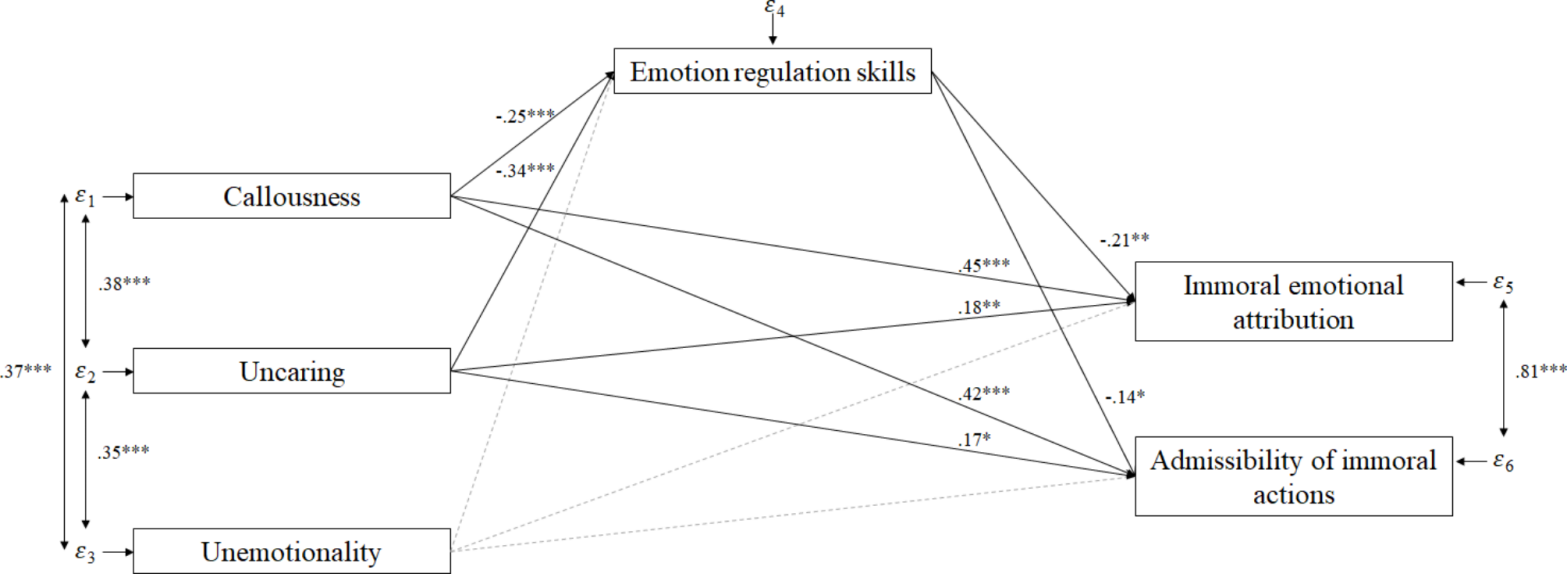
Path model with direct effects. *Note*. *p < .05; **p < .01; ***p < .001; non-significant paths were marked with gray dashed lines; gender, age and special educational need in emotional-social development were added as control variables

The tested path model revealed an adequate model fit (*χ²*(*df* = 1) = 3.55; *p* = .06; *CFI* = 1.00; *NFI* = 1.00; *TLI* = 0.86; *RMSEA* = 0.12, *SRMR* = 0.02). Figure 1 displays the significant direct effects and Table 2 displays the path coefficients of all tested direct effects. In the model, 41% of the variance of emotion regulation skills, 43% of the variance of immoral emotional attribution, and 40% of the variance of admissibility of immoral actions was explained. Regarding the direct effects, only the dimensions callousness and uncaring were negatively related to emotion regulation skills, and positively related to immoral emotional attribution and admissibility of immoral actions. No significant direct effects emerged for the unemotionality dimension. Emotion regulation skills were negatively related to immoral emotional attribution and to admissibility of immoral actions. Gender had an effect on immoral emotional attribution, and age had an effect on the callousness, uncaring, and unemotionality dimensions. Special educational needs in emotional-social development had an effect on the callousness and unemotionality dimensions, and on emotion regulation skills. The indirect mediation effects are shown in Table 3. There were positive indirect effects between the callousness and uncaring dimension onto immoral emotional attribution, mediated by emotion regulation skills. Additionally, there was a positive indirect effect between the callousness and uncaring dimension onto admissibility of immoral actions mediated by emotion regulation skills.

**Table 2:** Direct effects of the tested path model

Dependent Variables	Independent Variables	Path coefficient (*β*)	*p*
Emotion regulation skills ←	Callousness	− 0.25	< 0.001
	Uncaring	− 0.34	< 0.001
	Unemotionality	− 0.12	0.059
	Gender	− 0.07	0.249
	Age	0.08	0.217
	SEN	− 0.22	< 0.001
Immoral emotional attribution ←	Callousness	0.45	< 0.001
	Uncaring	0.18	0.008
	Unemotionality	− 0.02	0.737
	Emotion regulation skills	− 0.21	0.002
	Gender	− 0.13	0.021
	Age	− 0.03	0.634
	SEN	− 0.05	0.404
Admissibility of immoral actions ←	Callousness	0.42	< 0.001
	Uncaring	0.17	0.013
	Unemotionality	0.11	0.103
	Emotion regulation skills	− 0.14	0.049
	Gender	− 0.10	0.072
	Age	− 0.03	0.684
	SEN	− 0.09	0.143
Callousness ←	Gender	0.08	0.256
	Age	0.15	0.047
	SEN	0.20	0.008
Uncaring ←	Gender	− 0.02	0.758
	Age	0.16	0.041
	SEN	0.01	0.859
Unemotionality ←	Gender	0.03	0.700
	Age	0.16	0.033
	SEN	0.17	0.026

**Table 3:** Indirect mediation effects between callous-unemotional traits scales and immoral emotional attribution and admissibility of immoral actions mediated by emotion regulation skills

	Path coefficient (*β*)	Bootstrap SE	95% Bootstrap confidence interval	
			Lower bounds	Upper bounds
Immoral emotional attribution^a^ ←				
Emotion regulation skills^b^				
Callousness^c^	0.08**	0.05	0.02	0.19
Uncaring^c^	0.10**	0.04	0.04	0.19
Unemotionality^c^	0.03	0.02	0.00	0.08
Admissibility of immoral actions^a^ ←				
Emotion regulation skills^b^				
Callousness^c^	0.05*	0.04	0.01	0.13
Uncaring^c^	0.07*	0.03	0.02	0.14
Unemotionality^c^	0.02	0.02	0.00	0.06

*Note*. *p < .05; **p < .01; ***p < .001, ^a^dependent variable, ^b^mediator, ^c^independent variable

## Discussion

Aligning with the overall aim to examine the associations between callous-unemotional trait dimensions, emotion regulation skills, and cognitive and emotional aspects of morality in middle childhood, a mediational path-analysis was conducted. Our findings align with our hypotheses concerning the direct association from emotion regulation skills onto morality (H2), as well as the mediation by emotion regulation skills between callous-unemotional traits and morality (H4). Hypotheses concerning the callous-unemotional trait dimensions were only partially supported, as callousness and uncaring had direct effects onto emotion regulation skills (H1) and morality (H3), but not the unemotionality dimension.

Considering the first hypothesis (H1), we found that the dimensions callousness and uncaring were negatively associated with emotion regulation skills. Hence, children with higher levels of callousness and uncaring, also report lower levels of emotion regulation skills. Similarly, correlations between callous-unemotional traits and emotion regulation have been found in other studies [43, 100, 101]. Yet due to a lacking theoretical framework and longitudinal studies that explore the developmental pathways, authors have examined various connections, including moderating interactions between callous-unemotional traits and emotion regulation [100], comparison of emotion regulation between children with and without conduct disorders and callous-unemotional traits [102], and even emotion regulation as a predictor for callous-unemotional traits [11].We proposed that callous-unemotional traits would impact children’s social-emotional development, and thus their acquired emotion regulation skills. However, the current study draws upon cross-sectional data, and thus inferences about the directional causality of callous-unemotional traits and emotion regulation cannot be made. Longitudinal studies that explore causality within the development of the examined constructs can be used to build upon the concurrent associations found in the current study. Given the different etiological underpinnings of callous-unemotional traits, scholars have proposed distinguishing between two callous-unemotional trait profiles [103]. Primary callous-unemotional traits result from genetic/biological deficits in emotion processing, whilst secondary callous-unemotional traits result from an emotional deficit brought about by pathogenic environmental factors [103]. Emotion processing and recognition have been found to differ between the two profiles [102], and future research should be conducted to examine whether the development of adequate emotion regulation skills and strategies is impeded for both.

Considering the second hypothesis (H2), we found that emotion regulation skills were negatively associated with immoral emotional attribution and admissibility of immoral actions. Thus, the higher the emotion regulation skills were, the less likely children are to associate positive feelings with immoral decisions, i.e., they are more likely to link immoral decisions with feeling bad. Furthermore, the higher the emotion regulation skills were, the less likely children are to view immoral acts as permissible, i.e., they are more likely to consider the actions unacceptable. These findings are consistent with previous research and provide preliminary indications that emotion regulation skills may be conducive for children’s morality [30–33]. Possessing emotion regulation skills can ensure that individuals feel empathy and focus on the emotions of others, which is vital in moral situations [7, 10, 30]. When presented with moral conflicts, opting for the moral choice is associated with personal costs (e.g., being late for class, if taking the time to help someone). Hence, making the moral choice within moral conflicts is not only accompanied by positive feelings (e.g., pride) but also negative feelings, such as distress [18, 104]. Difficulties in regulating distress costs cognitive resources [30, 32], yet may be easier for children with higher emotion regulation skills. Glazer [105] proposed that moral emotions are carefully regulated states, which only promote cooperative behaviors when adequately developed in childhood. Adequately developed emotion regulation skills thus promote moral emotions and cooperation, whilst inadequate emotion regulation skills result in unrestrained moral emotions that discourage cooperation [105]. In their Social Information Processing-Moral Decision-Making Framework, Garrigan et al. [10] also proposed that emotion processes should be seen as a central component, influencing all steps in moral decision making, from encoding and interpreting cues to moral response decision and behavior; Garrigan et al., [10] include emotion regulation as one of the important emotion processes. Our findings reiterate the importance of emotional processes, and provide further evidence that emotion regulation skills should be seen as a significant component in understanding morality.

Regarding the third hypothesis (H3), we found that the dimensions callousness and uncaring were positively associated with immoral emotional attribution and admissibility of immoral actions, whilst unemotionality was not. The results align with the general connection observed between callous-unemotional traits and morality [46–48, 106]. The current finding that children high in overarching callous-unemotional traits are more likely to associate positive feelings with immoral decisions, is also consistent with those of Pardini and Bryd [107], who showed that elementary school children with higher callous-unemotional traits were less likely to expect remorse in perpetrators of aggressive behavior, and reported lower empathic concern towards victims. Regularly being confronted with moral situations, children learn that moral transgressions can lead to negative emotions, such as guilt [108]. However, children with psychopathic traits show deficits in recognizing emotions [52] and emotional arousal [9], and higher levels of emotion suppression [103], which indicates that their emotional reactivity and responsiveness in moral transgressions may be impaired, thereby affecting the learning process of morality [109]. The current finding that children high in callous-unemotional traits view immoral actions as more permissible, aligns with those of Vasconcelos et al. [49], who found that adolescents with higher callous-unemotional traits evaluate moral transgressions as less wrongful. Thornberg and Jungert [50] state that children with higher psychopathic traits may tend to be less concerned with the well-being of others and may therefore perceive moral transgressions as more permissible in the absence of explicit rules prohibiting it [47, 48]. The findings indicate that callous-unemotional traits can be incorporated into the Social Information Processing-Moral Decision-Making Framework [10].

In line with our fourth hypothesis (H4), indirect effects demonstrate that emotion regulation skills mediate the association between callous-unemotional traits (callousness and uncaring) and immoral emotional attribution and admissibility of immoral actions. The current findings indicate that children with high levels of callous-unemotional traits (specifically callousness and uncaring), possess lower emotion regulation skills, which in turn impacts immoral emotional attribution and admissibility of immoral actions. Reduced emotion regulation skills, thus detail one mechanism by which children with callous-unemotional traits respond to moral conflicts and transgression. This finding aligns with related studies, which revealed that emotion regulation difficulties mediate the association between overarching callous-unemotional traits and antisocial behaviors in young adults [110], and emotion dysregulation mediates the relationship between psychopathic traits and aggression in an adult sample [111].

Through the inclusion of control variables, we found gender generally did not have an effect on the variables, whilst age had a direct effect on callous-unemotional traits, as previously shown in the literature [62]. We further found that children with special educational needs in emotional-social development had higher levels of callousness and unemotionality, lower emotion regulation skills, yet did not differ in their immoral emotional attribution and admissibility of immoral actions from children without special educational needs. Future research could explore whether callous-unemotional traits predispose children to emotional and behavioral tendencies that increase the likelihood of receiving the status of special educational needs in emotional-social development. Furthermore, authors have suggested that students with special educational needs (especially in emotional-social development) could be at risk for atypical moral development [73, 112], thus the lack of effect on immoral emotional attribution and admissibility of immoral actions should be explored further.

### Limitations

A methodological limitation of the current study is that the German version of the Emotion Regulation Checklist [81] has not yet been psychometrically evaluated; yet reliability coefficients and EFA in the current study were sufficient/good. Furthermore, an ad hoc sample was recruited, and data collection was suspended before 300 children participated; nonetheless, the power analyses showed that the sample was sufficiently large (see Methods section). Although four of the model fit indices showed good values, the *RMSEA* value was quite high and the *TLI* somewhat low; this could be due to the complexity of the model. The callous-unemotional trait dimension of unemotionality neither had a direct effect on emotion regulation skills, nor on immoral emotional attribution and admissibility of immoral actions, despite significant correlations (see Table 1). This could indicate a redundancy effect [113, 114], with dimensions explaining the same variance or stronger direct effects prevailing, yet future research is needed to clarify.

### Practical implications and conclusion

Morality is a complex, multifaceted construct, with multiple existing theories [1] and numerous influencing individual, contextual, and societal factors [7, 8]; this may have contributed to the paucity of preventions and interventions specifically targeting development of morality. As affective components, such as empathy and affective concern, are conducive for motivating morally relevant actions [7, 10], focusing on such factors may be a good starting point. Targeting emotion regulation skills is promising, as it also affects morality [30–33], can be targeted and modified in school contexts [115, 116]. The results of the present study support previous findings that highlight the importance of emotion regulation skills in promoting social conflict skills and preventing aggression [101, 117, 118]. Prevention and intervention programs should promote social-cognitive as well as emotion-related skills so that children are able to solve social problems [117–119]. Targeting factors that have been found to influence both callous-unemotional traits and emotion regulation skills, such as parenting dimensions and attachment [43, 102, 120, 121], may be another option to support the development of morality.

## Data Availability

The datasets used and/or analysed during the current study are available from the corresponding author on reasonable request.
